# Increased Duodenal Eosinophil Degranulation in Patients with Functional Dyspepsia: A Prospective Study

**DOI:** 10.1038/srep34305

**Published:** 2016-10-06

**Authors:** Lijun Du, Jinhua Shen, John J. Kim, Yunxian Yu, Liqin Ma, Ning Dai

**Affiliations:** 1 Department of Gastroenterology, Sir Run Run Shaw Hospital, Zhejiang University, Hangzhou, Zhejiang province 310016, China; 2 Division of Gastroenterology, Loma Linda University, Loma Linda, CA 92354, USA; 3 Department of Epidemiology and Health Statistics, School of Public Health, Zhejiang University, Hangzhou, Zhejiang province 310058, China; 4 Department of Pathology and Pathophysiology, Zhejiang University, Hangzhou, Zhejiang province 310058, China

## Abstract

Functional dyspepsia (FD) is a functional gastrointestinal disorder diagnosed by symptom-based criteria. It has been said that duodenal immune activation plays a role in the pathogenesis of FD. The primary aims of the study were to compare the total number of duodenal eosinophil and evaluate the eosinophil degranulation rate, number of duodenal degranulated eosinophil and mast cell between patients with FD and healthy subjects. We enrolled 96 patients with FD and 24 healthy controls at Sir Run Run Shaw Hospital. The total number of eosinophil was comparable in the second portion of duodenum (D2) and duodenal bulb (D1) between patients with FD and healthy controls (all *P* > 0.05). Significant higher eosinophil degranulation positive rate in D2 (*P* = 0.003) and a trend towards higher in D1 (*P* = 0.084) were observed in patients with FD compared with healthy controls. Moreover, the number of duodenal degranulated eosinophil in patients with FD were significantly increased than healthy controls in D1(9.8 ± 6.3 vs 2.9 ± 2.1 per HPF, *P* = 0.0002) and a trend towards increase in D2 (10.7 ± 7.7 vs 5.3 ± 0.9 per HPF, *P* = 0.077), respectively. However, degranulated mast cells in patients with FD were almost same with healthy controls. Increased eosinophils degranulation in duodenum play an important role in pathogenesis of FD.

Functional dyspepsia (FD) is a common functional gastrointestinal disorder impacting 20% of people worldwide and up to 24% of Chinese population^1^,^2^,^3^. Although FD does not affect survival, it does significantly impair quality of life and work productivity^4^,^5^. As the cause of FD is undetermined and satisfactory treatment is limited, patients usually undergo repeated medical examinations leading to substantial use of healthcare resources. Because of lacking structural lesions, FD is diagnosed by using symptom-based Rome III criteria, and characterized by symptoms of postprandial fullness, early satiation, epigastric pain and/or epigastric burning within the past 3 months with symptom onset at least 6 months earlier in the absence of any organic or structural disease^6^. There are two subcategories of FD: postprandial distress syndrome (PDS) and epigastric pain syndrome (EPS)^6^. FD is likely to represents a spectrum of heterogeneous conditions and the mechanisms of pathogenicity are poorly understood. There has been a large focus on altered gastro-duodenal motility, gastro-duodenal hypersensitivity to gastric distention, *Helicobacter pylori (H. pylori*) infection, and psychological disturbance^7^,^8^,^9^,^10^,^11^,^12^,^13^,^14^. Currently, there is increasing evidence for the presence of low-grade immune activation in the duodenal mucosa of patients with FD^15^,^16^,^17^. Holtmann *et al.* found patients with FD have significantly increases in systematic cytokines such as TNF-α, IL-1β, and IL-10 compared with healthy controls^18^. Furthermore, mast cells and eosinophils are hypothesized to be involved in the pathogenesis of FD and may contribute to the development and persistence of gastrointestinal symptoms^15^,^19^,^20^. For example, patients with FD have increased eosinophil and mast cell infiltration in the duodenum which may be indicative of ongoing low-grade inflammation^21^. In a population-based case-control study, a higher proportion of patients with FD had ≥22 eosinophil count (5 non-overlapping high powered fields, HPFs) was observed compared to asymptomatic controls in the first portion (AOR = 11.7, 95% CI 3.9–34.9) and the second portion (AOR = 7.3, 95% CI 2.9–18.1) of the duodenum^15^. However, in a study of 136 Japanese patients with FD, duodenal eosinophilia were limited to patients with post-infectious FD^22^. In another Australian study of FD, only patients with post prandial distress syndrome and early satiety subtypes demonstrated elevated duodenal eosinophil count compared to asymptomatic controls, and no increase of mast cell number was observed^17^,^23^. Finally, a case-control study of 25 Iranian FD patients that compared to 27 asymptomatic controls did not demonstrate any association between FD and duodenal eosinophilis and mast cells^24^.

The association between FD and duodenal eosinophil and mast cell counts were inconsistent. Some studies were limited by small sample size or comorbid with other functional gastrointestinal disorders. To further investigate the hypothesis, the aims of the study were to: (1) compare the total number of gastro-duodenal eosinophil between patients with FD and healthy subjects in Chinese population; (2) evaluate the eosinophil degranulation positive rate, number of duodenal degranulated eosinophil and mast cell between patients with FD and healthy subjects; (3) evaluate associations of specific FD subtypes or symptoms and *H. pylori* infection status with the number of gastro-duodenal eosinophils.

## Methods

This was a prospective case-control study comparing the total number of gastro-duodenal eosinophil and degranulated eosinophils between patients with FD and healthy individuals.

### Participants

We consecutively recruited patients newly diagnosed FD, and asymptomatic controls who were scheduled to undergo upper gastrointestinal endoscopy as part of annual health examination or surveillance of gastrointestinal metaplasia at outpatient gastroenterology clinic of Sir Run Run Shaw hospital from March 2014 to May 2014. The criteria for inclusion were: (1) age between 18 and 70 years, (2) symptoms meeting Rome III criteria for patients with FD and absence of dyspeptic symptoms for control, and (3) unremarkable endoscopic findings. Patients with FD were further assessed for symptoms severity and categorized to PDS and EPS groups according to Rome III criteria^25^. The criteria for exclusion were: (1) progressive, severe diseases requiring active medical management (e.g. advanced cardiac, liver, renal or neurological disease, advanced cancer), (2) medical conditions known to increase peripheral and tissue eosinophilia (inflammatory bowel disease, celiac disease, vasculitis, connective tissue disease, hypereosinophilia syndrome, active infection and transplantation), (3) atopic disease such as asthma, allergic rhinitis, and eczema, and (4) history of significant gastrointestinal pathology (gastro-esophageal reflux disease and peptic ulcer disease) and history of gastrointestinal surgery (except appendicectomy, cholecystectomy, hernia repair). The ethical committee of Sir Run Run Shaw Hospital approved this study (No. 20140304-6). All participants signed the informed consent prior to enrolling in this study. All the protocols were carried out in accordance with the approved guidelines.

### Abdominal symptom questionnaires

All participants completed a group of questionnaires contained basic characteristics, Hospital Anxiety and Depression Scale (HADS), and simplified abdominal symptom questionnaire, which contains the frequency and severity of abdominal symptoms.

### Histopathologic analysis

All recruited participants underwent upper gastrointestinal endoscopy, and routine examination of blood, stool ovum, serological total IgE, and anti-tissue transglutaminase antibody (tTG-IgA). *H. pylori* infection was determined by positivity of both C13 breath test and gastric histology. Experienced endoscopists performed all upper gastrointestinal endoscopies. Biopsy specimens were collected from lesser curvature of gastric body, lesser curvature of gastric antrum, duodenal bulb (D1), and second portion of duodenum (D2). Then biopsies were fixed in 10% formalin and processed to paraffin embedding for hematoxylin and eosin (HE) staining by routine methods. Five non-overlapping fields on the slides (400× magnification, Olympus) were randomly selected and quantified by two independent observers (JS, LM), to determine the total number of gastro-duodenal eosinophils (HE staining) and expressed per 5 HPFs.

### Eosinophil and mast cell immunostaining

Immunohistochemistry (IHC) stains were used to evaluate the expression of major basic protein (MBP) and tryptase, which are markers of eosinophil and mast cell degranulation, respectively^26^. After dewaxing and rehydrating, tissue sections (D1, D2) were treated with 0.3% hydrogen peroxide in methanol to block endogenous peroxidase activity followed by pepsin or heat-mediated antigen retrieval. After blocking with 3% goat serum for 20 minutes at room temperature to minimize nonspecific staining, the sections were incubated with mouse anti-eosinophil MBP antibody (1:50, AbD Serotec, UK) and mouse anti-mast cell tryptase antibody (1:26000, Abcam, USA) for 1 hour at room temperature. After washing, the sections were treated with HRP-labeled goat anti-mouse IgG (ZSGB, China) for 30 minutes. The reaction was visualized using diaminobenzidine (DAB kit, ZSGB, China) and hematoxylin staining. Eosinophil degranulation positive was defined by the presence of MBP granules stain in randomly selected fields. If there was evidence of eosinophil degranulation on IHC stain, five non-overlapping fields on the slides (400× magnification, Nikon) were randomly selected and analyzed by two independent observers (LD, LM), to determine the mean numbers of degranulated eosinophil and mast cell and expressed per HPF.

### Statistical analysis

Data were presented as mean ± SD or median. Differences between groups were analyzed by Students’ t-test for normal distribution or Wilcoxon two-sample test for non-normal distribution. Categorical values were compared using Chi-square tests. The concordance rate of two independently observers’ eosinophil counting was assessed by concordance correlation coefficient test. The relationship between eosinophil counts and the symptoms or subgroups of FD were examined by linear regression model. Data were analyzed using SAS 9.4 and SPSS 22.0, and a two-tailed *P* < 0.05 was considered statistically significant.

## Results

### Study population

Ninety-six patients with FD and 24 healthy controls were included in this pilot study (64% female). Demographic and clinical characteristics of participants were described in Table 1. There were no significant differences in age, frequency of smoking, alcohol drinking, and education between two groups, as well as *H. pylori* positivity rate (*P* = 0.522). The body mass index (BMI) score in patients with FD was significantly lower than asymptomatic controls. While the anxiety and depression scores in patients with FD were significantly higher than healthy controls (*P* < 0.0001).

Serum total IgE of all patients and healthy controls were normal. The mean tTG-IgA value in patients with FD was 2.8(SD, ±1.4), compared with 4.2(SD, ±4.1) in controls, and no one was positive. The stool ovum detection of patients and controls were all negative. None of patients and healthy controls had celiac disease, acute gastroenteritis or obvious allergy.

### Reliability of eosinophil counting

There was moderate to good agreement of eosinophil counting, the correlation coefficients between two observers were 0.59, 0.65, 0.96 and 0.9 in D2, D1, gastric antrum and gastric body, respectively.

### Gastro-duodenal eosinophil counts in participants

Four sites (D2, D1, gastric antrum and body) of eosinophils were counted using HE staining. The results of gastro-duodenal eosinophil counts in participants were presented in Table 2. Patients with FD had mean eosinophil counts of 58.0 (SD, ±28.1) and 55.0 (SD, ±30.5) compared to 55.1 (SD, ±31.0) and 56.2 (SD, ±33.1) in healthy controls at D2 and D1, respectively. The eosinophil counts at D2 and D1 in two groups were quite similar (*P* = 0.66 and 0.86 respectively). The median eosinophil counts in patients with FD were 27.0(14.0–60.0) and 26.0 (12.5–46.0) compared with 20.5(9.5–65.3) and 19.5 (10.0–29.3) in controls at gastric antrum and body, respectively. There was also no significant difference of eosinophil counts at stomach between patients and controls (*P* = 0.39 and 0.22 respectively).

### Duodenal eosinophil and mast cell degranulation in participants

**Figure 1:**
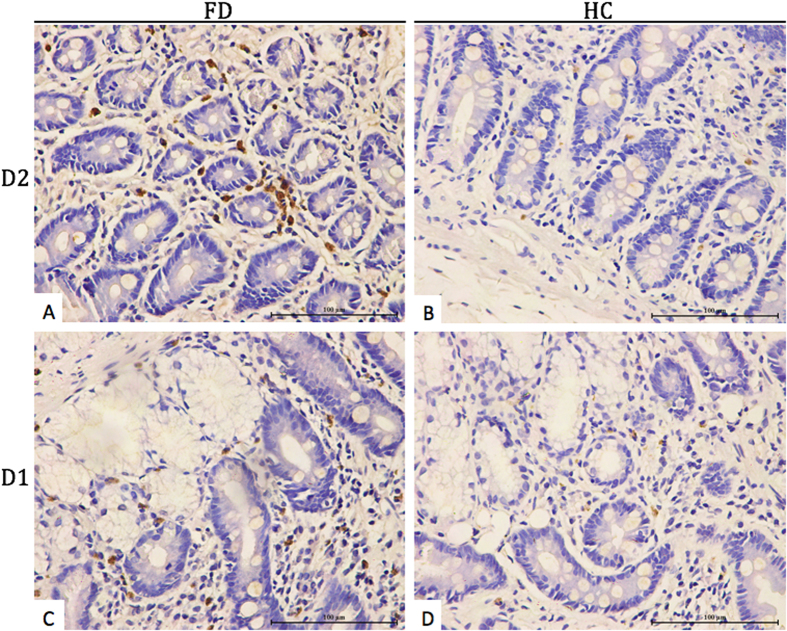
MBP immunostaining of eosinophil degranulation in the representative specimens (D2 and D1) from patients with FD and healthy controls. Magnification, 400×.

**Figure 2:**
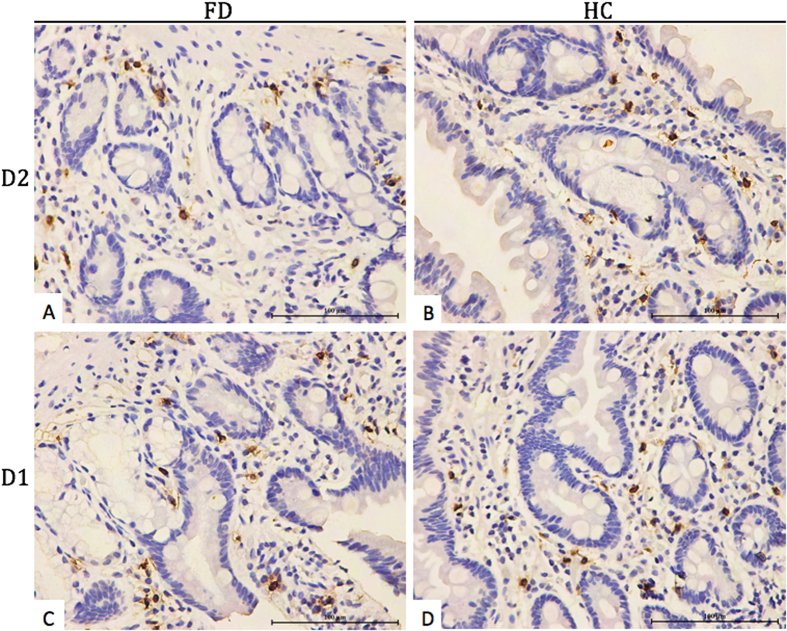
Tryptase immunostaining of mast cell degranulation in the representative specimens (D2 and D1) from patients with FD and healthy controls. Magnification, 400×.

Eosinophil degranulation was observed in 49 (59%) of 83 patients with FD, while 6 (25%) of 24 healthy controls (*P* = 0.003) in D2. Similarly, eosinophil degranulation was observed in 53 (64%) of 81 patients with FD, while 11 (46%) of 24 healthy controls (*P* = 0.084) in D1. Moreover, compared with healthy controls, the number of degranulated eosinophil in patients with FD showed an increased trend in D2 (10.7 ± 7.7 vs 5.3 ± 0.9 per HPF, *P* = 0.077, Fig. 1A,B) and significant increase in D1 (9.8 ± 6.3 vs 2.9 ± 2.1 per HPF, *P* = 0.0002, Fig. 1C,D). However, the number of degranulated mast cell in D2 (13.6 ± 2.9 vs 14.8 ± 2.4 per HPF, *P* = 0.242, Fig. 2A,B) and D1 (13.6 ± 2.7 vs 14.6 ± 2.7 per HPF, *P* = 0.292, Fig. 2C,D) of patients with FD were almost same with healthy controls. Detailed data were summarized in Table 3.

### Gastro-duodenal eosinophil counts and dyspeptic symptoms

Adjusting for age, BMI, alcohol drinking, and education, there was no association between presence and severity of early satiety and gastro-duodenal eosinophil counts (all *P* > 0.05). Nor did any positive relationship can be seen between postprandial fullness or epigastric pain and eosinophil counts in duodenum (all *P* > 0.05). However, there was a positive relationship between the frequency of fullness and eosinophil counts (β = 0.11, se = 0.05, *P* < 0.05) and a negative relationship between the frequency of epigastric pain and eosinophil counts (β = −0.09, se = 0.04, *P* < 0.05) in gastric body. Detailed data were listed in Table 4. There was no association between EPS or PDS and gastro-duodenal eosinophil counts (all *P* > 0.05).

### Gastro-duodenal eosinophil counts and *H. pylori* infection

*H. pylori* positive rate was 26.0% in patients with FD and 29.2% in healthy controls, respectively. Eosinophil counts were significantly related to *H. pylori* infection in gastric antrum and body in comparison with those who were not infected in both patient and control groups. No relationship was observed between *H. pylori* infection and duodenal eosinophil counts (all *P* > 0.05). Detailed data were summarized in Table 5.

## Discussion

Our study have demonstrated: (1) patients with FD were characterized by increased infiltration of degranulated eosinophils in duodenal mucosa compared to healthy controls with comparable total number of eosinophils (HE staining) in gastro-duodenal tract; (2) a positive relationship between frequency of fullness and eosinophil counts and a negative relationship between frequency of epigastric pain and eosinophil counts was observed in gastric body mucosa of patients with FD; (3) gastric eosinophil counts but not duodenal eosinophil counts are related to *H. pylori* infection. However, duodenal degranulated mast cells in patients with FD were not increased.

The mean eosinophil counts were 58.0 ± 28.1 and 55.1 ± 31.0 per 5HPFs in D2, and 55.0 ± 30.5 and 56.2 ± 33.1 per 5HPFs in D1 among patients with FD and healthy controls using HE staining, respectively. The duodenal eosinophil counts among participants in the present study appear greater (58.0 ± 28.1 vs 34.6 ± 16.9, *P* < 0.001 in D2 of patients; 55.1 ± 31.0 vs 18.6 ± 10.5, *P* < 0.001 in D2 of controls) than those reported among participants from an Australian cohort^15^. Given these unexpected differences, evaluation for causes of tissue eosinophilia (stool ova and parasite, serum IgE) were performed for all the subjects in the study and an experienced pathologist reviewed all the tissue sections to verify accuracy of the eosinophil count. Different lifestyle and dietary factors may account for the difference in the number of eosinophil counts between Chinese and Western populations. Food-induced immune activation in functional gastrointestinal disorders (FGIDs) has been previously recongized^27^. There is a possibility that genetic factors also account for this difference^28^. However, our study did not demonstrate a difference in eosinophil counts between patients with FD and healthy controls.

In order to further investigate whether increased degranulated eosinophils or mast cells play a role in pathogenesis of FD, we performed immunostaining of MBP and tryptase. Our results demonstrated eosinophil degranulation in higher proportion of patients with FD (59% vs 25%, *P* = 0.003) compared to healthy controls in D2. Similarly, a trend towards higher proportion with eosinophil degranulation was observed among patients with FD (64% vs 46%, *P* = 0.08) compared to healthy controls in D1. Furthermore, the number of degranulated eosinophils in duodenum were significantly increased in patients with FD ((9.8 ± 6.3 vs 2.9 ± 2.1 per HPF, *P* = 0.0002) in D1 and a trend towards increase (10.7 ± 7.7 vs 5.3 ± 0.9 per HPF, *P* = 0.08) in D2 compared to healthy controls. These findings suggest that activated eosinophils may be the dominant inflammatory mechanisms involved in the pathogenesis of dyspeptic symptoms. However, the present study does not support the role of degranulated mast cells in the pathogenesis of FD.

Parallel to findings in irritable bowel syndrome (IBS), emerging studies demonstrate that low-grade inflammation play a role in the pathophysiology of FD^19^,^21^,^29^. A meta-analysis suggested that nearly 12% of individuals who acquire an acute infectious gastroenteritis go on to develop FD suggesting that inflammation may be an important initiating factor in the development of FD^30^. Furthermore, histological studies showed that macrophage and eosinophil are associated with dyspeptic subtypes in patients with post-infectious FD^22^.

Mast cells and eosinophils in the duodenum are the two major inflammatory cells widely studied in FD. Duodenal hypersensitivity to acid and lipid exposure and subsequent dyspeptic symptoms have indicated the duodenum is a targeted pathogenic site in patients with FD^8^,^31^. Eosinophil-mast cell-nerve interactions are thought to play an important role in the generation of dyspeptic symptoms^15^. Although our study failed to demonstrate an association, others have demonstrated the importance of activated mast cells in FD^32^. Tryptase released from degranulated mast cells in association with PAR-2 receptor induce epithelial breakdown, promote immune activation, and propagate visceral hypersensitivity^33^,^34^,^35^. Mast cell is also a trigger for eosinophil migration into the gastrointestinal tract^36^. There is convincing evidence that mast cell tryptase activates eosinophils through PAR-2 receptor expressed on eosinophils^37^,^38^.

Early clinical evidence supporting the role of duodenal eosinophila in the pathogenesis of FD showed that 71% of children with FD exhibited duodenal eosinophila^39^. Montelukast, a competitive antagonist of cys LT_1_ receptor, also used for treatment of eosinophilic gastroenteritis, was found to relieve dyspeptic symptoms^40^. Other studies in adult FD patients have demonstrated that duodenal eosinophila as a promising biomarker and may be a unique feature of this condition^15^,^16^,^17^.

Eosinophil granules contain an array of cytotoxic granule proteins, including MBP, eosinophil cationic protein (ECP), eosinophil-derived neurotoxin (EDN), and eosinophil peroxidase (EPO)^41^. MBP, as the predominant granule protein, has been shown to modulate muscarinic receptor dysfunction, leading to smooth muscle contraction and gastric dismotility^42^,^43^. MBP also has been implicated in degranulation of mast cells and regulation of peripheral nerve plasticity that influence visceral sensitivity^41^,^44^. Furthermore, eosinophils can release a variety of compounds, including cytokines, chemokines, and neuromodulators^41^. Eosinophil-derived leukotrienes are triggers for eliciting upper gastrointestinal symptoms^45^. Building on previous work, our results suggest that increased eosinophil degranulation in duodenum play an important role in the pathogenesis of FD.

Besides, we have found gastric eosinophils infiltration have a positive linkage to fullness and a negative linkage to epigastric pain in present study. Previous study has reported a significant negative correlation between gastric emptying rate and severity of abdominal pain in children with FD^46^. Eosinophil and its activation in stomach inducing inflammation-neuron interaction may be the explanation for these observations. However, the underlying detailed mechanisms for this observation remains unknown, which needs basic research for further investigation.

The limitations of present study should be addressed: (1) the small sample size of healthy controls may affect the validity of this study; (2) this is an observational study and the mechanisms of activated eosinophil regulating FD is undetermined in present study, Further exploration of degranulated eosinophil induced development of FD and the way interaction between eosinophil and mast cell are in progress.

In conclusion, our findings indicated that the degree of duodenal degranulated eosinophils may be a better indicator of FD process rather than total number of eosinophil. There may provide important implications for management of dyspeptic symptoms.

## Additional Information

**How to cite this article**: Du, L. *et al.* Increased Duodenal Eosinophil Degranulation in Patients with Functional Dyspepsia: A Prospective Study. *Sci. Rep.* **6**, 34305; doi: 10.1038/srep34305 (2016).

## Figures and Tables

**Table 1:** Demographic and clinical characteristics of participants.

Variable	Case		Control		P value
	N	Mean ± S.D.	N	Mean ± S.D.	
Age, year	96	47.6 ± 11.2	24	45.2 ± 7.2	0.2025
Height, meter	96	1.6 ± 0.1	24	1.7 ± 0.1	**0.0109**
Weight, kg	96	58.8 ± 9.6	24	68.0 ± 13.2	**0.003**
BMI, kg/m^2^	96	22.2 ± 3.0	24	24.4 ± 3.3	**0.0022**
Anxiety score	96	6.2 ± 2.7	24	1.9 ± 1.2	**<0.0001**
	**N**	**Median**(**Q**_**25**_**~Q**_**75**_)	**N**	**Median**(**Q**_**25**_**~Q**_**75**_)	
Depression score	24	1(0~3)	24	0 (0~0)	<0.0001*
		**N** (**%**)		**N** (**%**)	
Smoking	No	15(15.6)		6(25.0)	0.28
	Yes	81(84.4)		18(75.0)	
Drinking	No	17(17.7)		8(33.3)	0.092
	Yes	79(82.3)		16(66.7)	

*Non-parameter analysis (Wilcoxon Two-Sample Test) was used.

**Table 2:** Total Gastro-duodenal eosinophil counts of participants (5HPFs).

Variable	Case		Control		P value
	N	Mean ± S.D.	N	Mean ± S.D.	
D2	96	58.0 ± 28.1	24	55.1 ± 31.0	0.656
D1	96	55.0 ± 30.5	24	56.2 ± 33.1	0.860
	**N**	**Median**(**Q**_**25**_**~Q**_**75**_)	**N**	**Median**(**Q**_**25**_**~Q**_**75**_)	
Antrum*	96	27.0(14.0~60.0)	24	20.5(9.5~65.3)	0.387
Body*	96	26.0(12.5~46.0)	24	19.5(10.0~29.3)	0.223

*Non-parameter analysis (Wilcoxon Two-Sample Test) was used.

**Table 3:** Degranulated eosinophil and mast cell counts of participants (HPF).

Variable	Case		Control		P value
	N	Positive rate (%)	N	Positive rate (%)	
D2 (EOS)	83	49 (59.0%)	24	6 (25.0%)	**0.003**
D1 (EOS)	81	53 (65.4%)	24	11 (45.8%)	0.084
	**N**	**Mean ± S.D**	**N**	**Mean ± S.D.**	
D2 (EOS)	49	10.7 ± 7.7	6	5.3 ± 0.9	0.077
D1 (EOS)	53	9.8 ± 6.3	11	2.9 ± 2.1	**0.0002**
D2 (MC)	83	13.6 ± 2.9	24	14.8 ± 2.4	0.242
D1 (MC)	81	13.6 ± 2.7	24	14.6 ± 2.7	0.292

**Table 4:** Associations of dyspeptic symptoms (and severity) with gastro-duodenal eosinophil counts.

Location	Fullness	FLevel	EarlyS	ELevel	Pain	PLevel
β (se)						
D2	1.49(1.24)	0.62(3.25)	−1.83(1.01))	−4.42 (2.48)	0.04(1.00)	1.73(2.68)
D1	0.32(1.43)	−1.21(3.72))	−1.25 (1.17)	−2.15(2.88))	−0.84(1.13)	−3.12(3.06)
Ln(antrum)^#^	0.05(0.05)	0.04 (0.13)	−0.07 (0.04)	−0.17(0.10)	0.01(0.04)	0.07(0.11)
Ln(body)^#^	**0**.**11**(**0**.**05**)	0.11(0.13)	−0.02 (0.04)	−0.02 (0.10)	**−0**.**09**(**0**.**04**))	−0.19(0.11))

*Adjustment for age, BMI, Smoking, Drinking, Education, occupation and family location.

^#^The value was transferred by nature log.

^$^P value <0.05.

^$$^FLevel, ELevel, PLevel meant the severity of fullness, early satiety and epigastric pain.

**Table 5:** Associations of *H. pylori* infection with gastro-duodenal eosinophil counts.

Dyspepsia	HP Infection	N	Mean ± S.D.	β(se)	P
Descending portion of duodenum					
Yes	Yes	25	49.04 ± 28.01	Ref	.
	No	71	61.15 ± 27.66	4.1(6.1)	0.5015
No	Yes	7	55.86 ± 39.54	Ref	.
	No	17	54.75 ± 28.25	−12.25(8.57)	0.1531
Duodenal bulb					
Yes	Yes	25	44.88 ± 21.03	Ref	.
	No	71	58.51 ± 32.56	4.47(6.98)	0.5224
No	Yes	7	53.89 ± 38.07	Ref	.
	No	17	57.17 ± 32.03	−5.28(5.67)	0.3513
Ln(Gastric antrum)#					
Yes	Yes	25	3.91 ± 0.81	Ref	.
	No	71	3.1 ± 0.94	−0.82(0.22)	**0.0003**
No	Yes	7	3.96 ± 0.72	Ref	.
	No	17	2.8 ± 0.97	−0.93(0.27)	**0.0005**
Ln(Gastric body)#					
Yes	Yes	25	3.42 ± 1.21	Ref	.
	No	71	3.03 ± 0.93	−0.53(0.24)	**0.0277**
No	Yes	7	3.73 ± 0.83	Ref	.
	No	17	2.62 ± 0.94	−0.98(0.37)	**0.0078**

^*^Adjustment for age, BMI, Smoking, Drinking, Marriage, Education, occupation and hhhhhfafamily location. Transferred by nature log.

^#^The value was transferred by nature log.
