# Corrigendum: Increased Duodenal Eosinophil Degranulation in Patients with Functional Dyspepsia: A Prospective Study

**DOI:** 10.1038/srep46121

**Published:** 2017-04-07

**Authors:** Lijun Du, Jinhua Shen, John J. Kim, Yunxian Yu, Liqin Ma, Ning Dai

Scientific Reports 6: Article number: 3430510.1038/srep34305; published online: 10 06 2016; updated: 04 07 2017

Affiliation 1 was incorrectly listed as ‘Department of Gastroenterology, Sir Run Run Shaw Hospital, Zhejiang University, Hangzhou, Zhejiang province 310016, China’ in the original version of the Article. The correct affiliation is listed below:

Department of Gastroenterology, Sir Run Run Shaw Hospital, School of Medicine, Zhejiang University, Hangzhou, Zhejiang province 310016, China.

This has now been corrected in the HTML and PDF versions of this Article.

